# Increased photocorrosion resistance of ZnO foams via transition metal doping†

**DOI:** 10.1039/d2ra06730g

**Published:** 2023-01-17

**Authors:** Zachary Warren, Jannis Wenk, Davide Mattia

**Affiliations:** a Department of Chemical Engineering, University of Bath UK d.mattia@bath.ac.uk

## Abstract

ZnO is a widely studied photocatalyst, but practical use is hindered by its low resistance to photocorrosion in water, which leads to metal leaching and loss of performance over time. In this work, highly porous and mechanically stable ZnO foams, called MolFoams, were doped by adding 1% or 2% Co, Ni or Cu salts to the starting Zn salt, followed by air insufflation during a sol–gel rection and sintering. The resulting doped foams showed a major increase in stability, with a 60–85% reduction in Zn^2+^ leaching after irradiation, albeit with a reduction in photocatalytic activity. A systematic analysis using XRD, Raman, XPS and XANES allowed for the identification of dopant species in the foams revealing the presence of Co_3_O_4_, NiO and Cu_2_O within the ZnO lattice with doping leading to a reduced band gap and significant increases in the resistance to photocorrosion of ZnO while identifying the cause of the reduction in photocatalytic activity to be shifting of the band edge positions. These results provide a pathway to significantly reduce the photocorrosion of ZnO in water, with further work required to maintain the photocatalytic activity of undoped ZnO.

## Abstract 2

Practical use of ZnO is hindered by its low resistance to photocorrosion in water, which leads to metal leaching and loss of performance over time. Here we demonstrate how metal doping can significantly increase ZnO's resistance to photocorrosion.

## Graphical abstract

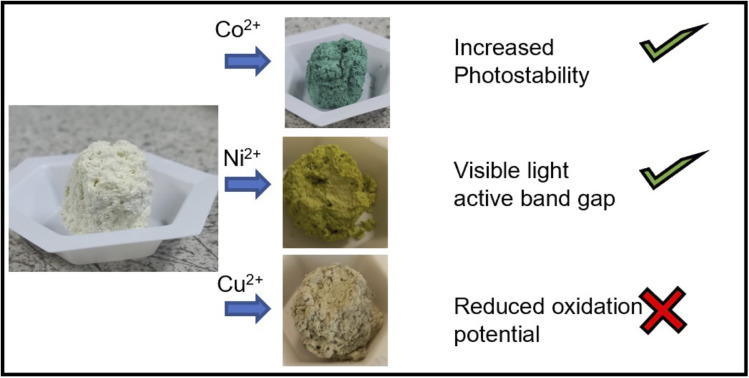

## Introduction

ZnO is well reported for its use as a photocatalytic material in water treatment.^1^ Its beneficial properties include high electron mobility and low toxicity, while also absorbing over a wider range of wavelengths of light than widely used TiO_2_, allowing for greater utilisation of light.^2^

However a significant drawback of ZnO is its susceptibility to photocorrosion under UV irradiation in an aquatic environment,^3^ leading to the dissolution and formation of Zn^2+^ ions in solution as shown in eqn (1a) and (1b).^4^1a
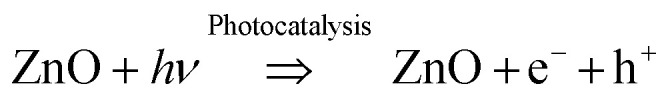
1b
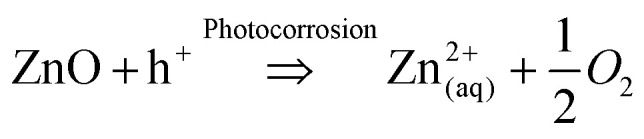


Given the UV-induced production of photogenerated holes, higher intensities of incident light impinging on the photocatalyst lead to increased photocorrosion of ZnO.^3^ Hence, it is essential to assess photocatalytic activity in conjunction with photocorrosion resistance in the case of ZnO. Dissolved oxygen content also plays a role in the photocorrosion of ZnO, as in oxygen deficient conditions, the progress of photocorrosion leads to the generation of oxygen from the ZnO structure.^5^ While it has been shown that operation under oxygen saturated conditions can reduce the impact of photocorrosion,^4^ considerations still need to be made regarding its use in water treatment as the World Health Organisation limits the maximum concentration of Zn^2+^ in water to 3.0 ppm.^6^

Additionally, ZnO is a wide band gap semiconductor, with a band gap of 3.2 eV, meaning that only short wavelength, ultra-violet irradiation can be utilised for the degradation of organic pollutants. Furthermore ZnO suffers from a relatively high surface recombination of charge carriers (e^−^/h^+^) further reducing its photocatalytic activity.^7^

Doping of semiconductors is a long-established method for adapting and improving photocatalyst materials.^8^ Doping with both metals and nonmetals leads to red-shifted band gaps and visible light active photocatalysts.^9^ Metal doping introduces shallow or deep level states, above the valence band or below the conduction band, allowing for the absorption of longer wavelengths of light, or the formation of heterojunctions due to the coupling of semiconductors with different bandgaps, leading to greater charge separation and e^−^/h^+^ lifetimes due to a reduction in recombination rates.^10^ However, it has been reported that the presence of the mid-gap level states show the potential to act as recombination centres, particularly when care is not taken to avoid the formation of metal clusters.^11^

ZnO is a promising candidate for doping to further improve the photocatalytic activity under visible light irradiation. As an intrinsic n-type semiconductor, the presence of p-type dopants, often transition metals, leads to the formation of an acceptor level below the original conduction band of ZnO, narrowing the band gap.^11^ Furthermore, through selective use of transition metals that form oxides with a valence band higher in energy than ZnO, e^−^/h^+^ charge pair separation can be achieved, with the holes moving to the valence band of the dopant metal from the ZnO.^12^ This leads to a twofold benefit, increased charge separation, which is key to improving efficiency of photocatalytic systems,^13^ and the removal of h^+^ from the ZnO surface, which reduces the photocorrosion and dissolution of Zn^2+^ into solution.^3^

The state of the art for ZnO doping shows a wide range of potential dopants, from non-metals such as C, N, or S,^14–16^ to a wide range of metals including, transition metals,^17^ and rare earth metals.^18^ However these procedures have only been applied to films or particles of ZnO and as such are still hindered by the low surface areas and the need for downstream removal when applied in water treatment.^19,20^

MolFoams, or Molecular Foams, is the term used to describe foams produced using the synthetic method herein and developed previously.^21^ Through the use of a sol–gel synthesis with controlled incorporation of air to produce a porous structure which is then sintered to produce a singular interconnected foam structure made of metal oxide without the presence of discrete particles.

In this work doping of ZnO foams was achieved *via* addition of dopant metal salts to an aerated sol–gel solution. The photocorrosion resistance was based on dissolved Zn content in solution after photocatalysis and assessed using ICP-MS while photocatalytic activity measured through analysis of the degradation of carbamazepine, an anticonvulsant and target micropollutant.^22^

This work was conducted to apply the improvements in photocatalytic activity and stability seen only in doped ZnO films and particles to foams, to be used in a system that shows promise for wide-scale adoption due to overcoming the limitations of slurry and immobilised catalysts.

## Experimental

### Materials

Zinc acetylacetonate (Zn(AcAc)_2_; ≥95.0%), cobalt acetylacetonate (Co(AcAc)_2_; ≥99.0%), nickel acetylacetonate (Ni(AcAc)_2_; ≥99.0%), copper acetylacetonate (Cu(A0cAc)_2_; ≥99.0%), oxalic acid anhydrous (≥99.9%), hexadecyltrimethylammonium bromide (CTAB; ≥99.9%), polyethylene glycol (PEG; 10 000), carbamazepine and ethanol (absolute) were all purchased from Sigma Aldrich and used as provided. Jacketed, fritted funnels were purchased from Chemglass Lifesciences and fitted with PTFE sheets (Zwanzer). Desiccant from a Drierite™ gas-drying unit (Sigma-Aldrich) was used as provided by the manufacturer but transferred to a smaller tube.

### Synthesis of doped foams Zn_1−*x*_M_*x*_O

Doped ZnO foams were synthesised using a modified method from previous work.^21^ Briefly, M^2+^(AcAc)_2_ (15.0 mmol) was added to a 25 mL Pyrex beaker with M^2+^(AcAc)_2_ being a combination of Zn(AcAc)_2_ and the acetylacetonate salt of the dopant metal (Co, Ni, Cu), such that the total molarity of metal salt in the reaction solution was kept constant at 15 mmol. Exact masses and molar quantities are shown in Table 1. Subsequently, 15 mL of a 10 mM CTAB solution was made up in ethanol and added to the beaker. Oxalic acid (15.0 mmol) and 40 μmol PEG 10 000 with 10 mL EtOH were mixed in a separate beaker. Both solutions were stirred at 60 °C for 60 minutes in an oil bath until homogenous solutions were obtained. The metal acetylacetonate solution was added to a PTFE-lined, temperature controlled jacketed filter funnel at 60 °C, followed immediately by the oxalic acid solution. The reaction mixture was aerated with compressed air with an upward flow rate of 0.1 standard litres per minute (sL min^−1^) using a rotameter.

**Table 1:** Molarities and masses of metal salts used in synthesis of doped ZnO MolFoams

Dopant		Metal salt				
Dopant % (*x*)	mmol dopant	Co(AcAc)_2_	Ni(AcAc)_2_	Cu(AcAc)_2_	Zn(AcAc)_2_	
		Dopant mass/g			mmol	Mass/g
0.5	0.075	0.0193	0.0193	0.0196	14.925	3.9344
1	0.150	0.0386	0.0385	0.0393	14.850	3.9147
2	0.300	0.0771	0.0771	0.0785	14.700	3.8751

The reaction mixture of the zinc/dopant and acid solutions was aerated for 3 hours leading to the formation of a coloured gel, red/pink for cobalt-doped, light green for nickel-doped and blue for copper-doped samples. The gel was then transferred to a pre-weighed ceramic crucible and placed in a preheated muffle furnace (Carbolite CWF 1100) at 80 °C and dried for 12 hours to remove any remaining ethanol, resulting in a dry doped zinc oxalate foam which was stored under ambient conditions.

Conversion of doped zinc oxalate foams into doped zinc oxide foams and removal of remaining organic components was achieved using a two-step thermal sintering process: The zinc oxalate foam was sintered using a furnace, heated to 1000 °C with a ramp rate of 5 °C min^−1^ and held at temperature for 0.5 hours, and then 900 °C with a ramp rate of 5 °C min^−1^ and held at temperature for 20 hours. This resulted in the formation of a mechanically stable doped ZnO foam. The high temperature sintering was also used to remove any remaining organic components. After sintering, the foams were cylindrical in shape, with an average diameter of 20 ± 1 mm and height of 19 ± 1 mm.

### Characterisation of doped foams Zn_1−*x*_M_*x*_O

The surface morphology of the doped foams was studied using a JEOL JSM-7900F FESEM. Prior to imaging, samples were coated with 20 nm Cr. The crystal structure of the foams was investigated using a STOE STADI P dual powder transmission X-ray diffractometer using a scanning range of 2*θ* = 20–90° and a scan time of 20 minutes. The chemical stability of the MolFoams was analysed using inductively coupled plasma mass spectrometry (ICP-MS) in a Thermo Fisher Scientific X-Series II instrument. All samples, standards, and blanks were spiked with internal standard elements Be, In, and Re. The Zn, Co, Ni and Cu concentrations were calibrated using six synthetic standards prepared from a 1000 ppm Inorganic Ventures (VA, USA) standard. The associated error was typically lower than 1.0%.

Measurements of the porosity of the MolFoam were conducted using the Archimedes principle:^23^1
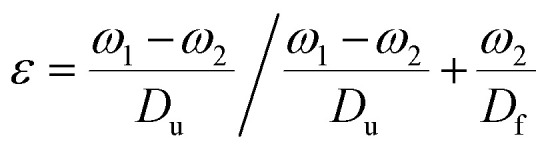
where *ε* is the porosity of the foam, *ω*_1_ is the mass of the wet foam, *ω*_2_ is the mass of the dry foam, *D*_u_ is the density of water (deionised, ultrapure) and *D*_f_ is the density of ZnO.

UV-Vis spectroscopy for surface characterisation was conducted using a PerkinElmer LAMBDA 650s series spectrometer. The reflectance of the doped and undoped ZnO produced in this work and the data gathered to conduct the band gap analysis was collected using a UV/Vis/NIR spectrometer with an integrating sphere.

Raman spectra used in this work were collected using a Renishaw InVia Confocal Raman microscope, excitation laser wavelength 532 nm, 100% laser power at 74 mW on the sample with 2.6 s exposure time, and a diffraction grating of size 1800 I/mm with slit opening of 65 μm. Detector used was a 1040 × 256 pixel CCD camera.

XPS and UPS data was acquired using a Kratos Axis SUPRA using monochromated Al Kα (1486.69 eV) X-rays at 15 mA emission and 12 kV HT (180 W) and a spot size/analysis area of 700 × 300 μm and a He(i) UV lamp running at 20 mA emission. The instrument was calibrated to gold metal Au 4f (83.95 eV) and dispersion adjusted give a BE of 932.6 eV for the Cu 2p3/2 line of metallic copper. Ag 3d5/2 line FWHM at 10 eV pass energy was 0.544 eV. Source resolution for monochromatic Al Kα X-rays is ∼0.3 eV. The instrumental resolution was determined to be 0.29 eV at 10 eV pass energy using the Fermi edge of the valence band for metallic silver.

XANES analysis was performed on an easyXAFS 300+ spectrometer, with Ag or Mo X-rays operating at 40 mA and 15 kV emission and silicon spherical bent crystal analysers.

### Photocatalytic reactor setup

The photocatalytic activity of the doped MolFoams was analysed using a bespoke recirculating reactor, reported previously.^21^ Reactor cartridges were made up of a quartz tube (*h* = 250 mm, OD = 25 mm, ID = 22 mm) with a 3D printed plastic buffer designed to hold the foams in place and prevent loss of the foam into the tubing and pump, positioned to avoid interference with the light source.

MolFoams of known mass (0.7 g) were placed inside the cartridge and secured using Subaseal fittings, connected to a gear pump (Ismatec, MCP-Z with a pump head Model GBS.P23.JVS.A-B1, Cole Parmer) and to a jacketed beaker of 500 mL (acting as the reservoir) with a magnetic stirrer, where the temperature was maintained using a water-cooled bath (RC-10 Digital Chiller, VWR). Three UV lamps (Aquatix pond UV lamp, *λ* = 254 nm, 5 W), positioned equidistant around the quartz tube reactor at a distance of 3 cm, served as the light source.

### Photocatalytic activity (PCA) experiments

PCA experiments were conducted using 10 μM solutions of carbamazepine (CBZ) in 500 mL unbuffered ultrapure water at 10 ± 1 °C. CBZ was selected as a model organic micropollutant for photocatalytic activity (PCA) studies, due to its high UV stability,^24^ known degradation pathways^25^ as well as allowing for comparison with prior work conducted on MolFoams.^21^ To minimize photocorrosion of ZnO,^4^ CBZ solutions were saturated with O_2_ for 40 minutes prior to experiments. The recirculating reactors were operated at flow rate of 250 mL min^−1^. Control experiments were conducted in the absence of MolFoams in the reactor both under irradiation and in the dark.

For all photocatalysis experiments, CBZ removal was monitored from 1 mL aliquots collected during sampling every 15 minutes for the first hour and every 30 minutes thereafter, such that the total volume removed was less than 10% of the starting reservoir volume, using high performance liquid chromatography (HPLC).

HPLC analysis of CBZ was performed on a Thermo Scientific Ultimate 3000 liquid chromatograph with a UV detector. CBZ analysis used a Thermo Scientific Acclaim 120 C18 column (3.0 × 75.0 mm, particle size 3.0 μm) and a Thermo Scientific Acclaim 120 C18 guard column (*R*) 120 C18 (3.0 × 10.0 mm, particle size 5.0 μm) The mobile phase was 5.0 mM phosphoric acid and acetonitrile 70 : 30 (v/v) with a flow rate of 0.8 mL min^−1^, injection volume of 20 μL and detection wavelength of 285 nm. Degradation of carbamazepine was measured *via* plotting (*C*_*t*_/*C*_0_) *vs.* time where *C*_0_ is the initial concentration of CBZ and *C*_*t*_ is the concentration of CBZ at a given time. The pseudo first order degradation kinetics (*k*) was calculated *via* linear regression of a plot of ln(*C*_*t*_/*C*_0_) *vs.* time.

### Photocatalyst quantum yields

The quantum yield (*Φ*) of a photocatalytic system is defined as the number of molecules of pollutant (carbamazepine) undergoing degradation relative to the number of photons reaching the catalyst surface.^26^ The photon flux (*E*_qf_) arriving at the surface of the photocatalyst along with the kinetic constant (*k*) allows calculating the quantum yield, assuming negligible photon loss due to scattering and all photons are absorbed by the photocatalyst. Details of the calculations are provided in the ESI.†

### Electrical energy per order (EEO)

To assess the scale-up potential of the system, the energy consumption of the reactor was estimated *via* the electrical energy per order (EEO), defined as the kilowatt hour of electrical energy needed to decrease the concentration of a pollutant by an order of magnitude (90%) in one cubic metre of solution:^27^2
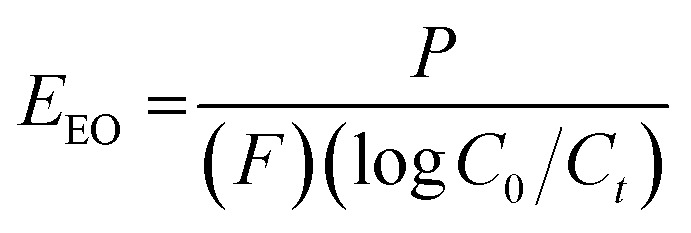
where: *P* is the power output of the lamps and *F* is the volumetric flow rate of solution. Details of the calculations are provided in the ESI.†

## Results and discussion

### Characterisation of doped MolFoams

**Fig. 1:**
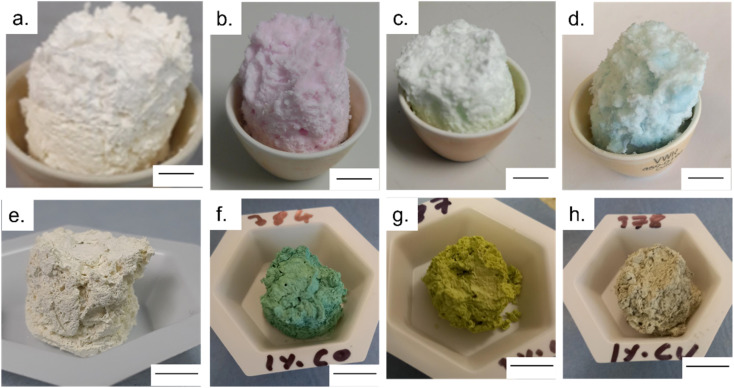
(a–d) images of dried Zn_1−*x*_M_*x*_O foams were M is Zn, Co, Ni or Cu, respectively; (e–h) images of sintered Zn_1−*x*_M_*x*_O foams were M is Zn, Co, Ni or Cu, respectively. Scale bar is 1 cm in all images.

Once removed from the funnel, the doped zinc oxalate monoliths were free standing and plastic under gentle compression and coloured according to the dopant metal: cobalt-doped foams were red/pink, nickel-doped foams were light green, and copper-doped foams were blue. The dried monoliths were 28 mm in diameter and 30 mm in height on average. This decreased to 20 ± 1 mm diameter and 19 ± 1 mm height post sintering and the foams were robust enough to be handled and subjected to flow experiments. Furthermore, in all cases a colour change occurred during the sintering of the doped foams, with the cobalt-doped turning to a deep green, the nickel-doped foams becoming yellow, and the copper doped turning grey, indicating the formation of the respective metal oxides,^28–30^ as seen in Fig. 1. The gravimetric porosity of the foams was calculated using the Archimedes principle^23^ and found to be 95 ± 2%, comparable with aero- or xero-gels without the need for supercritical solvent extraction nor volatile foaming agents.^31,32^

**Fig. 2:**
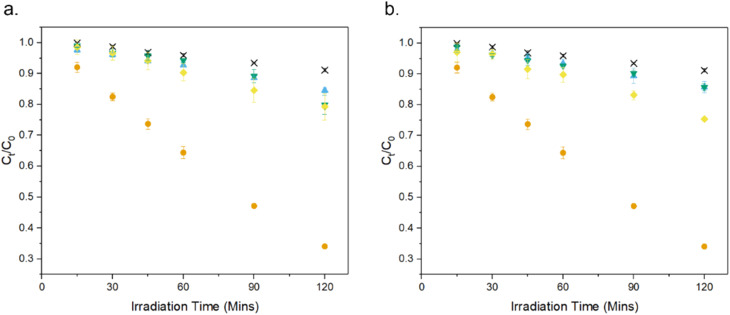
Photocatalytic degradation of CBZ using ZnO doped with (a) 1% and (b) 2% of various transition metals: × photolysis, ● undoped, ▼ Co,◆ Ni, ▲ Cu. Numerical values provided in Table 2.

The photocatalytic activity of the MolFoams was investigated by analysing the degradation of carbamazepine in a recirculating flow reactor operated at a previously optimized flowrate of 250 mL min^−1^.^21^ In the absence of irradiation, removal of carbamazepine *via* adsorption was found to be negligible. When the reactors were operated without MolFoam, the degradation due to photolysis was found to be minimal (9%) after 2 hours of irradiation. Undoped ZnO MolFoams provided strong photocatalytic activity and increased the degradation of carbamazepine to 66% (Fig. 2). The incorporation of dopant into the ZnO structure led to significant decreases in photocatalytic activity with foams doped with 2% Ni showing the lowest decline, at 25% removal of carbamazeping over the same irradiation time. Comparing dopant metals at 1% concentration shows there is no significant difference in photocatalytic activity when using different metal dopants, while when doping increases to 2%, the Ni doped foams show greater photocatalytic activity than those doped with Co or Cu, but still significantly lower than the pure ZnO foams. Fig. S1† provides an alternative data presentation arranged by dopant metal. Co-doped foams showed reduced activity at higher dopant %, Ni-doped foams showed an increase in activity with dopant concentration, while Cu-doped foams showed no significant difference in activity. This is in contrast to reported findings for doped ZnO slurries in the literature, which in the case of Co, Ni and Cu, show increasing the dopant concentration leads to an increase in photocatalytic activity, up to around 5% at which point activity plateaus or decreases.^33–35^

As expected by the reduced photocatalytic activity, the doped foams had significantly lower quantum yield and higher electrical energy per order (*E*_EO_) values, both indicating the foams were less efficient in terms of electrical energy and photon usage, as shown in Table 2.

**Table 2:** CBZ removal for pure and doped ZnO MolFoams, pseudo first order degradation kinetics (*k*) and Zn concentration post photocatalytic degradation. Also tabulated are quantum yield (*Φ*) and EEO for each condition

Sample	*C*_120_/*C*_0_	*k* (×10^−3^)/min^−1^	[Zn^2+^]/ppb	[Co]/ppb	[Ni]/ppb	[Cu]/ppb	*E*_EO_/kWh m^−3^	*Φ*	Ref.
ZnO	0.34 ± 0.01	9.08 ± 0.44	482	—	—	—	21	8.73 × 10^−4^	21
1% Co	0.80 ± 0.03	1.70 ± 0.12	193	13	—	—	105	1.40 × 10^−4^	This work
2% Co	0.85 ± 0.01	1.30 ± 0.05	99	16	—	—	145	1.31 × 10^−4^	
1% Ni	0.79 ± 0.04	2.02 ± 0.11	112	—	8	—	100	2.01 × 10^−4^	
2% Ni	0.75 ± 0.01	2.04 ± 0.11	80	—	5	—	82	1.20 × 10^−4^	
1% Cu	0.85 ± 0.01	1.36 ± 0.04	102	—	—	2	145	1.34 × 10^−4^	
2% Cu	0.85 ± 0.02	1.27 ± 0.05	71	—	—	2	145	9.68 × 10^−5^	

However, doping foams with different metals led to 60–85% less photocorrosion of ZnO compared to the undoped samples, as indicated by the lower Zn^2+^ concentrations measured in solution after irradiation experiments (Table 2), far in excess of reported findings on coupled photocatalyst films.^36^ One potential explanation for this reduction in photocorrosion, and why it decreases at increased dopant concentration irrespective of dopant, is that the presence of the dopant metals leads to changes in the crystal structure of ZnO, leading to decreases in the surface energy. This, in turn, would reduce the possibility of hole attack, resulting in less photocorrosion of ZnO.^37^ An alternative mechanism for the reduction in photocorrosion is charge separation of holes onto dopant atoms, thus reducing the attack of ZnO by holes, with higher dopant concentrations leading to a greater number of dopant atoms to allow for the greater charge separation.^38^ Additionally, the ICP-MS analysis of the solution post-photocatalysis, for the specific metal used (*i.e.* for the [Co] for the Co doped foams) can be seen in Table 2. In all cases the concentration of metals were significantly lower than the WHO limits for drinking water.^6^

The XRD patterns (Fig. S2†) in all cases showed the formation of hexagonal wurtzite ZnO with lattice parameters of *a* = *b* = 3.25 Å and *c* = 5.21 Å, with the strongest intensity for the peaks associated with the (100), (002) and (101) crystal phases, and with sharp peaks indicative of a highly crystalline structure. All peaks are in agreement with those reported from JCPDS no. 36-1451,^39^ as well as previously reported work on MolFoams. For all dopants and different dopant concentrations, these peaks showed slight broadening with an average increase in full width at height maximum (FWHM) of 5%, indicating a decrease in crystallinity of the samples. In all cases, XRD analysis showed single phase hexagonal wurtzite structure, indicative of doping through the crystal lattice.^40^ Furthermore, the absence of crystal phases of Co, Ni or Cu, nor mixed metal oxides, should be noted, indicating that no dopant metal or metal oxide clusters were observed in the samples. The three most intense peaks of the XRD pattern show peak shifting towards higher angles for both Ni and Cu doping, while only the (101) peak shows higher angle shifting with Co doping, indicating a reduction in the unit cell. This is consistent with the fact that the atomic radii of the dopant metals are all smaller than that of Zn^2+^.^41^ However, due to the low dopant concentrations and small peak shift, doping had no impact on the lattice parameters of the crystal. In the case of all dopants, the intensity of the (100), (002), and (101) peaks decreased as did the relative intensities of the (100)/(101) and (002)/(101) ratios with average reductions of 0.02 and 0.03 respectively. This is indicative of the increased presence of (1011) facets in the crystal lattice which have shown to be highly reactive and beneficial for photocatalysis.^42^ The decrease in crystallinity caused by doping of metal ions into the lattice, as observed by the broadening of the peaks, has been shown to lead to a decrease in the surface energy of the ZnO lattice.^43^ This decrease in surface energy has a stabilising effect on the ZnO, and reduces the susceptibility to attack from holes, thus leading to the observed increase in resistance to photocorrosion.

The FE-SEM micrographs show the presence of irregular pores surrounded by an interconnected network of crystallites, along with morphological features unique to each dopant: Undoped ZnO shows the presence of faceted rod-like crystals, reported previously for MolFoams synthesised using 10 mM CTAB solutions. MolFoams doped with Co show larger, jagged crystals with clear facets along their length, while foams doped with Ni form faceted spheroidal crystals, and Cu doping forms smooth spheroidal crystals (Fig. 2).

**Fig. 3:**
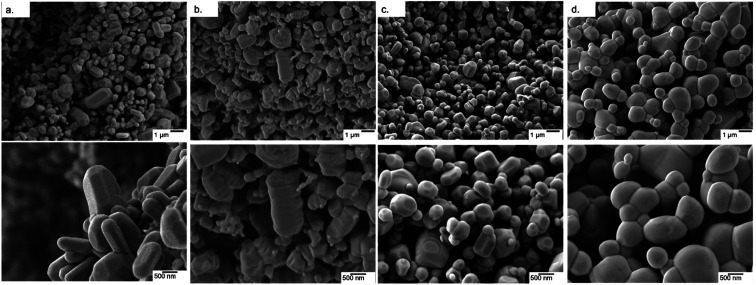
FE-SEM micrographs of (a) pure ZnO and ZnO doped with (b) Co, (c) Ni and (d) Cu at two different magnifications.

It is well-known that highly faceted crystal structures tend to be more photocatalytically active.^21^ As such, the smooth crystals obtained with copper doping (Fig. 3d) could explain the lower photocatalytic activity of these samples. However, the reduction in activity is too high to be attributed solely to the crystal morphology,^44^ and, in any case, this does not apply to Ni- and Co-doped foams due to the presence of faceted crystals seen in Fig. 3b and c. It is of note, the presence of faceted crystals in the doped samples is significantly reduced compared with the pure ZnO. While the XRD reports a slight decrease in the (100)/(101) and (002)/(101) ratios due to the presence of (1011) facets, the FESEM micrographs show the morphology to be smoother in all cases, particularly the Cu doped samples. This is of import, as the photocatalytically active facets also possess high surface energies, allowing for photocorrosion to occur as holes react at the surface.^45^ The reduction in the number of these faceted crystals leads to lower surface energies and as such, increased resistance to photocorrosion.

Additionally, elemental mapping confirmed uniform dispersion of dopant metal throughout the structure of the crystal lattice as shown in Fig. S3.†

**Fig. 4:**
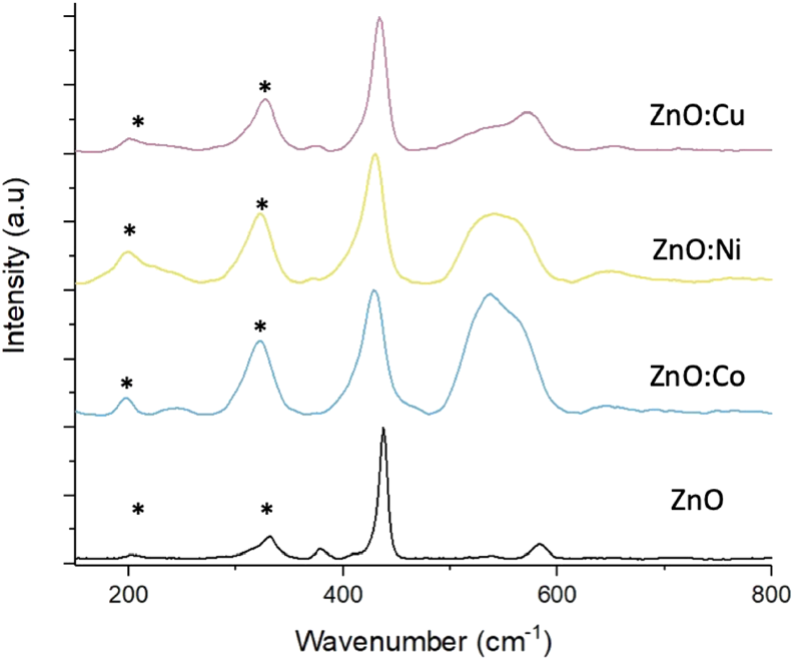
Raman spectra of pure and doped ZnO MolFoams. * correspond to multi-phonon features.

Raman scattering spectra were collected on both pure and doped ZnO samples to study the crystal lattice, particularly looking for phases of dopant metal oxide, present in potentially too low concentrations to be detected by XRD. Fig. 4 shows the Raman spectra from pure and doped ZnO samples collected with green excitation wavelength (532 nm). In all cases, the predominant peak corresponds to the non-polar optical phonon mode of ZnO, the high frequency *E*_2_ mode, associated with the vibrations pf oxygen atoms within the ZnO lattice.^46^ The spectra of the pure ZnO also shows the *A*_1_ and *E*_1_ Raman peaks at shifts of 379.6 and 583.9 cm^−1^ respectively. Peaks marked with an asterisk (*) are due to multi-phonon features present in the spectra and are caused by interaction between the *E*^low^_2_ peak, that can be detected at wavenumbers >100 cm^−1^, and the *E*2^high^ peak at 432.5 cm^−1^. Upon doping the ZnO, noticeable changes can be seen in the spectra. Firstly, downshifts occur for the *E*^high^_2_ and *E*_1_ peaks, which are attributed to increasing bond lengths between the zinc (or dopant) atom and oxygen atom in the lattice structure.^47^ Secondly, broadening of these peaks for all metal dopants, associated with surface disorder caused by incorporation of dopants into the lattice structure.^40^ As discussed earlier, this surface disorder and the reduced crystallinity leads to a reduction in photocorrosion due to lowered surface energies, that have been reported to be proportional to dopant concentrations.^48,49^

The broad peak present around 550 cm^−1^ in the Co and Ni doped samples is attributed to peak overlap between the weak ZnO peak, as seen in the pure sample, and a peak associated with either Co_3_O_4_,^50^ or NiO.^51^ CoO reports Raman peaks at around 675 and 455 cm^−1^, with the former being absent from the spectra, meanwhile Co_3_O_4_ exhibits Raman peaks in around 482, 519, 621 and 690 cm^−1^ with the peak at 519 cm^−1^ being clearly prominent in the spectra and increasing in intensity as doping concentration increases (Fig. S4†), while the peak at 482 cm^−1^ overlaps with the ZnO *E*^high^_2_ peak at 432.5 cm^−1^ leading to the peak broadening. The peak at 620 cm^−1^ can be seen in the doped samples and is absent from the pure, while the dopant concentration is too low to observe the peak at 690 cm^−1^. This suggests that the cobalt present in the doped foams is in the form of Co_3_O_4_ rather than CoO.

CuO exhibits an intense peak around 600 cm^−1^, which is absent from the spectra collected from the Cu doped foams. These samples instead exhibit a weak peak around 525 cm^−1^ which is used to characterise Cu_2_O rather than CuO,^52^ suggesting that the Cu within the doped foams is Cu(i) rather than the desired Cu(ii). The presence of Cu(i) only within the foams is also shown and corroborated by the XPS analysis (Fig. 6), and XANES analysis (Fig. S6†).

These effects are relative to the dopant concentration, with the higher dopant concentrations leading to greater peak shifting and broadening as well as the peaks associated with Co_3_O_4_ and NiO becoming more intense (Fig. S4†).

**Fig. 5:**
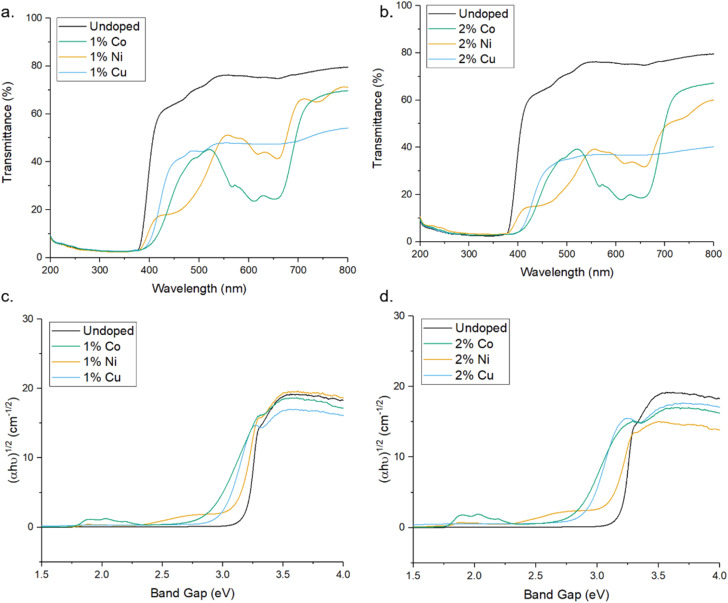
(a and b) UV-Vis transmittance spectra of pure and doped ZnO MolFoams. (c and d) show corresponding Tauc plots.

UV-Vis spectroscopy was used to evaluate the impact of doping transition metals on the optical properties of ZnO. Fig. 5 shows that pure ZnO exhibits strong absorption at 380 nm, corresponding to a band gap of 3.2 eV, further shown in the Tauc plots. For all dopant metals and concentrations, this absorption edge is red-shifted, corresponding to enhanced visible light response and a narrowing of the band gap. This is due to the formation of dopant levels in the band structure of ZnO, acting as acceptor levels below the conduction band, thus requiring lower energy photons to promote electrons from the valence band.^11^ Furthermore, all doped samples show greater absorbance across in the range of 400–800 nm compared to the pure samples, with cobalt and nickel doped samples exhibiting an increase in absorbance between 550 and 700 nm and the nickel exhibiting a broad absorption centred around 500 nm. These absorbances of specific wavelengths are responsible for the colour of the samples. By absorbing strongly in the region of ∼700 nm, associated with red light, the cobalt samples appear the complimentary colour of green. The same is true of the nickel doped samples, the absorption around ∼500 nm corresponds to violet light and as such the samples appear the complimentary yellow colour. The copper doped samples show no strong absorbance at a specific wavelength, instead absorbing over the entire visible range, leading to the grey colouration. These changes were clearly observable by eye, as all the doped samples were of darker colour than the white of the pure ZnO (Fig. 1).

**Fig. 6:**
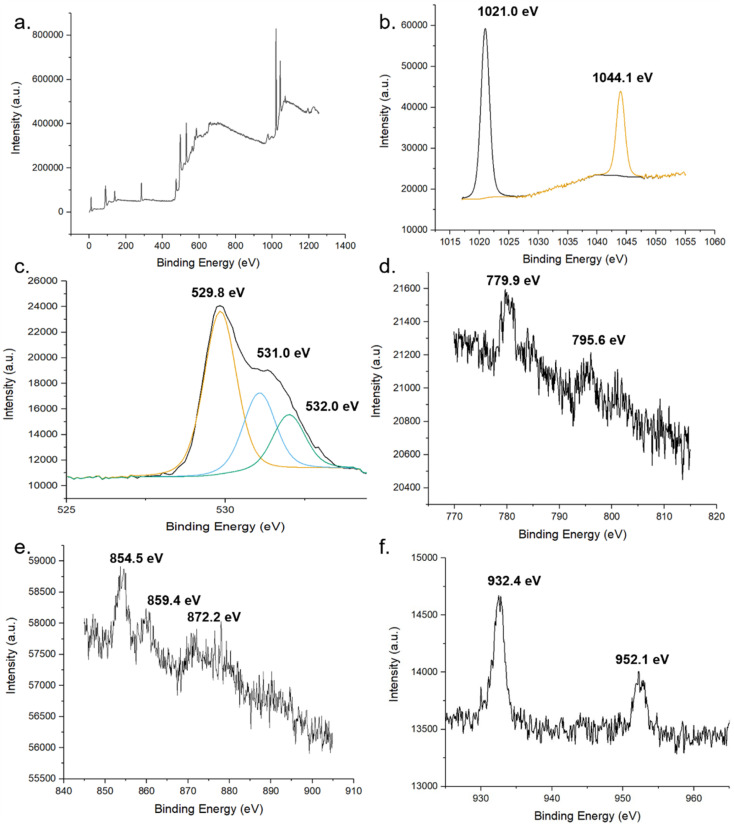
XPS spectra of pure and doped ZnO foams. (a) global, (b) Zn and (c) O of undoped foams. XPS spectra of 2p regions of TM dopant (d) Co, (e) Ni and (f) Cu.

The chemical states of compounds of the MolFoams were identified using the binding energies of the XPS spectra. The XPS provides further evidence for the incorporation of dopant into the ZnO lattice. Fig. 6 shows the XPS spectra of (b) Zn 2p, (c) O 1s and (d), (e) and (f) show the 2p spectra of Co, Ni, and Cu, respectively. In the Zn spectra, the two intense peaks at 1021.0 and 1043.9 eV are in agreement with the binding energies of Zn 2p_3/2_ and Zn 2p_1/2_ as well as the peak splitting of 23.1 eV confirming the Zn was present as Zn^2+^.^53^ This is further corroborated by the modified auger parameter (the sum of the kinetic energy of Zn auger peak and the binding energy of the Zn 2p_3/2_ peak) value of ∼2010 eV confirming the presence of ZnO rather than Zn metal.

The oxygen 1s peak was fitted by three components centred at 529.8, 531.0 and 532.0 eV. The low energy peak corresponds to O^2−^ ions in the wurtzite structure of ZnO, surrounded by Zn atoms with a full complement of nearest neighbour O^2−^ ions, such that the oxygen is in a fully oxidised stoichiometric environment.^54^ The highest energy peak is attributed to loosely bound oxygen species, often physisorbed water, at the surface of the sample.^40^ The medium binding energy component is attributed to O^2−^ in oxygen deficient regions of the ZnO lattice and is associated with oxygen vacancies within the crystal structure.^55^

In the Co-doped foams, despite the low doping concentrations, the two peaks corresponding to the Co 2p_3/2_ and Co 2p_1/2_ peaks at 779.9- and 795.6 eV respectively, in agreement with values reported elsewhere.^56,57^ Additionally the peaks show a spin-orbital splitting of 15.7 eV indicative of Co^2+^ and providing confirmation that no Co metal clusters are present in the samples as in this case the difference in peaks would be 15.0 eV.^40^

Furthermore, the region around the Co 2p_3/2_ peak shows a number of small peaks within the range of 779.9- to 785.5 eV including a broad peak centred around 780 eV. These peaks are attributed to Co_3_O_4_ which reports a number of peaks in this range, in particular peaks at 779.9- and 781.2 eV.^58^ Given the low concentration of dopant leading to low resolution, it is not possible to resolve these as separate peaks, and as such the spectra shows a single broad peak. This spectrum shows that while the dopant cobalt is only present within the lattice, with the absence of metal clusters at the surface, the cobalt exists in a mixed oxidation state as Co_3_O_4_ which is in agreement with the Raman (Fig. 4) and XANES (Fig. S6†).

Analysis of the Ni 2p XPS spectra main peak, associated with the 2p_3/2_ peak shows a doublet structure with peak splitting of ∼ 5 eV, likely caused by a metallic Ni 3d interaction with ligand oxygen 2p level in the valence band.^59^ Additionally, the position of the 2p_3/2_ peak and the 2p_1/2_ peak at 854.5- and 872.2 eV, respectively, are in good agreement with literature values reported for NiO.^60^

The XPS analysis of the Cu-doped foams clearly shows the presence of only Cu(i) species within the lattice, given the absence of satellite peaks in the Cu 2p spectra which would indicate the presence of Cu(ii).^53^ The presence of Cu(i) over Cu(0) is corroborated by the modified auger value as well as the shoulder region of the 2p_3/2_ peak, as Cu metal exhibits a sharp 2p_3/2_ peak while a shoulder region is reported in Cu(i), caused by trace amounts of Cu(ii) formed through self-oxidation.^61^ The presence of Cu(i) is further corroborated by the Raman spectroscopy.

These results are further corroborated by the XANES spectra (Fig. S6†) which confirm the presence of Co_3_O_4_,^62^ NiO,^63^ and Cu_2_O^64^ with the insets of Fig. S6a and c† showing the distinctive pre-edge features indicative of Co_3_O_4_, and Cu_2_O respectively.

These results further show that the substitution of cobalt, nickel and copper into the ZnO lattice can be achieved *via* simple modification of the previously reported MolFoam synthesis, leading to the formation of Co_3_O_4_, NiO and Cu_2_O respectively. Co_3_O_4_ has a visible light active band gap of 2.6 eV and has been shown to form a heterojunction with ZnO,^65^ and along with NiO,^66^ have been used as a photocatalyst, co-catalyst and a p type dopant and, as such, the presence of Co^2+^ and Ni^2+^ in the XPS spectra is desirable as these will lead to an increased charge separation of the holes, thus removing them from the ZnO surface, limiting the photocorrosion of ZnO. The increase in charge separation within ZnO doped with Co, Ni and Cu is well reported in the literature and shown experimentally *via* both photoluminescence and photoelectrochemical measurements.^67–70^ Photoluminescence studies have shown that an increase in dopant concentration leads to the formation of greater densities of oxygen interstitial and vacancy defects which act as traps for holes and electrons, respectively.^70^ This increased presence of charge trapping sites at higher dopant concentrations provides an explanation for the increased resistance to photocorrosion that is observed between the 1% and 2% samples for all dopant metals.

On the other hand, the presence of metal clusters could act as recombination centres, leading to a reduction in charge species lifetime and a subsequent reduction in photocatalytic activity.^71^

In the case of Cu doping, the presence of the Cu(i) rather than leading to increased charge separation, thus preventing photocorrosion of the ZnO, the Cu(i) acts as a species to be preferentially oxidised, as Cu(i) is highly susceptible to photocorrosion and oxidation to Cu(ii).^61^ The intent of the dopant was to reduce the photocorrosion of ZnO, without itself undergoing photocorrosion as overtime this will lead to the need to replace the photocatalyst, along with additional production of the foams. This is before consideration is placed on any potential reduction in activity caused by the formation of mixed oxide structures within the ZnO crystal lattice due to the irregular orientation and position within the lattice acting as recombination centres. Fig. S7† shows marginal decreases in photocatalytic activity when the concentration of Cu is increased from 0.5% to 1- or 2% highlighting this issue.

Furthermore, if it is found that the CuO provides greater beneficial effects on photocatalytic activity and reduction of photocorrosion, it is of interest that CuO be formed during the synthesis of the foams, rather than through gradual oxidation of Cu_2_O during the use of the photocatalyst.

**Fig. 7:**
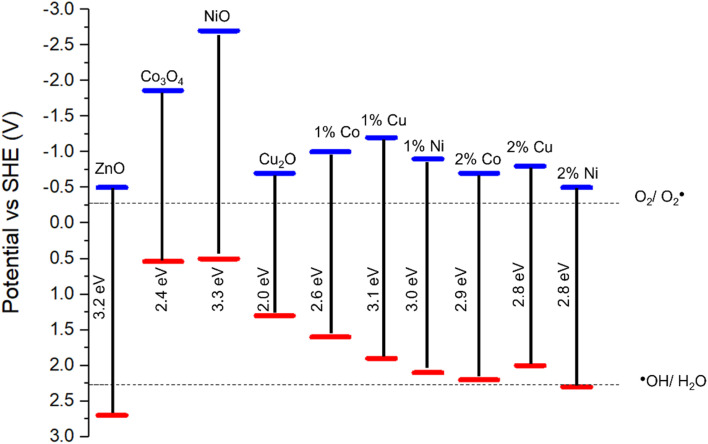
Band diagram of pure and doped ZnO based on UPS and Tauc plot calculations. Band edge positions for Co_3_O_4_, NiO and Cu_2_O obtained from literature.^76–78^

The impact of doping on the electronic structure was further elucidated using ultra-violet photoelectron spectroscopy (UPS), allowing for determination of the work function (*W*) and valence band maxima (VB_max_) of the materials which, when combined with the band gap, allows for the calculation of the conduction band minima (CB_min_) and study of the band edge positions of the photocatalyst.^72^Fig. 7 show the UPS spectra, along with valence band and cut-off regions of the spectra. The cut-off region allows for calculation of the work function (*W*) of the material, or the energy required to remove one electron from the material.^73^ Calculations and graphs of UPS spectra, including cut off and valence band regions can be found in the ESI.† From this value, and the intercept of the valence band region, the energy level of VB_max_ was calculated and, using the band gap derived from the Tauc plots in Fig. 4, a complete diagram of the band edge positions was constructed (Fig. 7). These calculations can be found in the ESI.† Inclusion of dopant metals at all concentrations leads to a lower binding energy in the valence bands of the material. This is caused by the formation of additional energy levels near or above the valence band due to the dopant metals being more electron deficient relative to Zn.^74^ Furthermore, the reduction in the band gap, as calculated through the Tauc plots, has a lesser impact on the band structure than the valence band edge, as the conduction band edge of the doped samples show less deviation from that of the pure sample, when compared to the valence band edges. The key information provided by the UPS is the band edge positions relative to the redox potentials for the reduction of oxygen into superoxide radicals. The oxidation of water into hydroxyl radicals are −0.28 V *vs.* standard hydrogen electrode (SHE) and +2.27 V *vs.* SHE respectively.^75^

For a photocatalyst to facilitate these redox reactions, the band edge positions must straddle the redox potential of both redox reactions to take place.^79^ As shown in Fig. 6g, in all cases except the ZnO doped with 2% Ni, the valence band edge is significantly lower that the redox potential for the ˙OH/H_2_O couple and, as such, lacks the oxidation capability required for the production of the hydroxyl radical from water, a key component in the photocatalytic degradation of organic compounds.^80^ The reduction in photocatalytic activity in the doped samples is likely caused by the increase in the valence band edge, reducing the oxidising potential of the foams and as such preventing the production of hydroxyl radicals for the degradation of carbamazepine. Given that the position of the 2% Ni doped foam band edge is only +0.03 V relative to the redox potential for the ˙OH/H_2_O couple, this provides an explanation as for why the activity of these samples were the highest among all doped foams but still significantly lower than pure ZnO which has a band edge position +0.43 V compared to the hydroxyl couple. Furthermore, this provides evidence the hydroxyl radicals are the active species for the degradation of organic pollutants as the band edge positions of the doped samples are positioned such that formation of hydroxyl radicals only is inhibited, whereas the band edge positions are still reducing enough to form superoxide radicals. At the same time as the photocatalytic activity of the doped samples are reduced compared to the pristine samples, indicating that the ability of the material to form superoxide radicals has minimal impact on the photocatalytic activity for the degradation of organic compounds when compared to hydroxyl radicals.

The inhibition of hydroxyl radical production of Co-, Ni and Cu-doped ZnO nanoparticles has been reported previously using the terephthalic acid photoluminescence technique along with a reduction in the photocatalytic activity of these samples relative to pure ZnO.^20^ Similar reductions in photocatalytic activity have been reported for Co- and Mn-doped ZnO nanoparticles with the reduction attributed to recombination centres.^81^ The dopant-induced shift in valence band edge position is highly significant and provides the best explanation for the low photocatalytic activities reported herein and within the literature.

## Conclusions

Building upon previous work on ZnO MolFoams, dopant metals were introduced into the foams to reduce the photocorrosion of ZnO, thus overcoming one of the key hindrances in its widespread application as a method for removal of recalcitrant organic micropollutants from water. Three different transition metals Co, Ni, and Cu, were doped into ZnO foams at concentrations of 1 and 2%, resulting in foams of a range of colours and crystal morphologies. Photocorrosion, as assessed by dissolved Zn content post degradation was reduced 60–85% in all doped foams and was attributed to increased charge separation induced by doping transition metals, leading to the formation of heterojunctions, as well as the doping induced disorder of the crystal structure reducing the surface energy of the material, reducing the susceptibility to attack from holes. Analysis of the structure of the doped foams was conducted to ascertain the impact of doping on the structure of ZnO as well as to identify the nature of the dopant species within the crystal structure. XRD, EDX, Raman and XPS were conducted and found that doping was successful and uniform throughout the structure with the cobalt doping leading to the formation of Co_3_O_4_, nickel doping forming NiO and copper-doped foams led to the formation of Cu_2_O species within the lattice. The cobalt and nickel doping lead to reduced photocorrosion through charge separation of holes, while the copper doping formed Cu_2_O which were preferentially oxidised to CuO by photocorrosion, rather than ZnO. In addition to specific effects caused by the individual dopants, all dopants lead to a decrease in crystallinity and a reduction in the surface energy of ZnO, further increasing the photocorrosion resistance.

However, doping of the foams lead to a decrease in photocatalytic activity. Advanced analytical methods were used to understand the cause of this loss in activity, with UPS and UV-Vis allowing for construction of a band edge diagram. This revealed that the doping of transition metal into the foams leads to a shift in the band edge positions, such that the foam no longer shows the required oxidation potential for the formation of the hydroxyl radicals necessary for the degradation of organic pollutants.

As such, further investigation is required into the impact of doping on the photocatalytic activity of ZnO, as the resistance to photocorrosion exhibited by the foams discussed here show promise for overcoming one of the key drawbacks of ZnO, as long as the photocatalytic activity is not hindered.

## Data availability

Data supporting this work is freely accessible in the Bath research data archive system at DOI: 10.15125/BATH-XXXX.

## Author contributions

Zachary Warren: conceptualisation, investigation, methodology, validation, visualisation, writing—original draft. Jannis Wenk: supervision, writing—review & editing. Davide Mattia: funding acquisition, project administration, resources, supervision, writing—review & editing.

## Conflicts of interest

There are no conflicts to declare.

## Supplementary Material

RA-013-D2RA06730G-s001
