# Correction: Increased photocorrosion resistance of ZnO foams *via* transition metal doping

**DOI:** 10.1039/d3ra90012f

**Published:** 2023-02-06

**Authors:** Zachary Warren, Jannis Wenk, Davide Mattia

**Affiliations:** a Department of Chemical Engineering, University of Bath UK d.mattia@bath.ac.uk

## Abstract

Correction for ‘Increased photocorrosion resistance of ZnO foams *via* transition metal doping’ by Zachary Warren *et al.*, *RSC Adv.*, 2023, **13**, 2438–2450, https://doi.org/10.1039/D2RA06730G.

The authors regret that the data availability statement was not correctly given in the original article. The correct statement is as shown below.

Data supporting this work is freely accessible in the University of Bath research data archive at DOI: 10.15125/BATH-01212.

The Royal Society of Chemistry apologises for these errors and any consequent inconvenience to authors and readers.

## Supplementary Material

