# Supplementary material for: Increased photocorrosion resistance of ZnO foams via transition metal doping
Source: RSC Adv. 2023 Jan 17;13(4):2438–50. doi: 10.1039/d2ra06730g (PMC9844254; doi:10.1039/d2ra06730g)
Supplement: RA-013-D2RA06730G-s001 [file RA-013-D2RA06730G-s001.pdf]

Electronic Supplementary Material (ESI) for RSC Advances.
This journal is © The Royal Society of Chemistry 2022

Supplementary Information for

Increased Photocorrosion Resistance of ZnO foams Through Transition Metal Doping.

Zachary Warren,[a] Jannis Wenk [a] and Davide Mattia*[a]

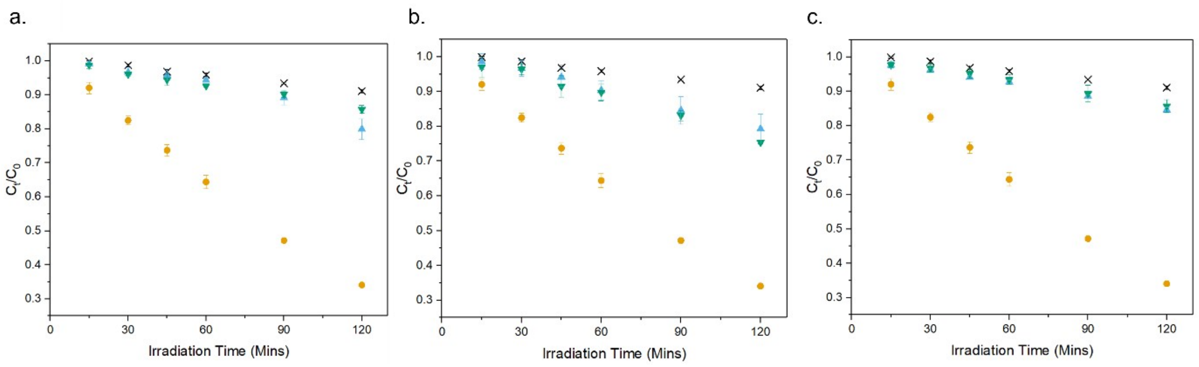

Figure S1: Photocatalytic degradation of CBZ using ZnO doped with various concentrations of a) Co, b) Ni and c) Cu: × photolysis, ● undoped, ◘ 1%, and ❑ 2%.

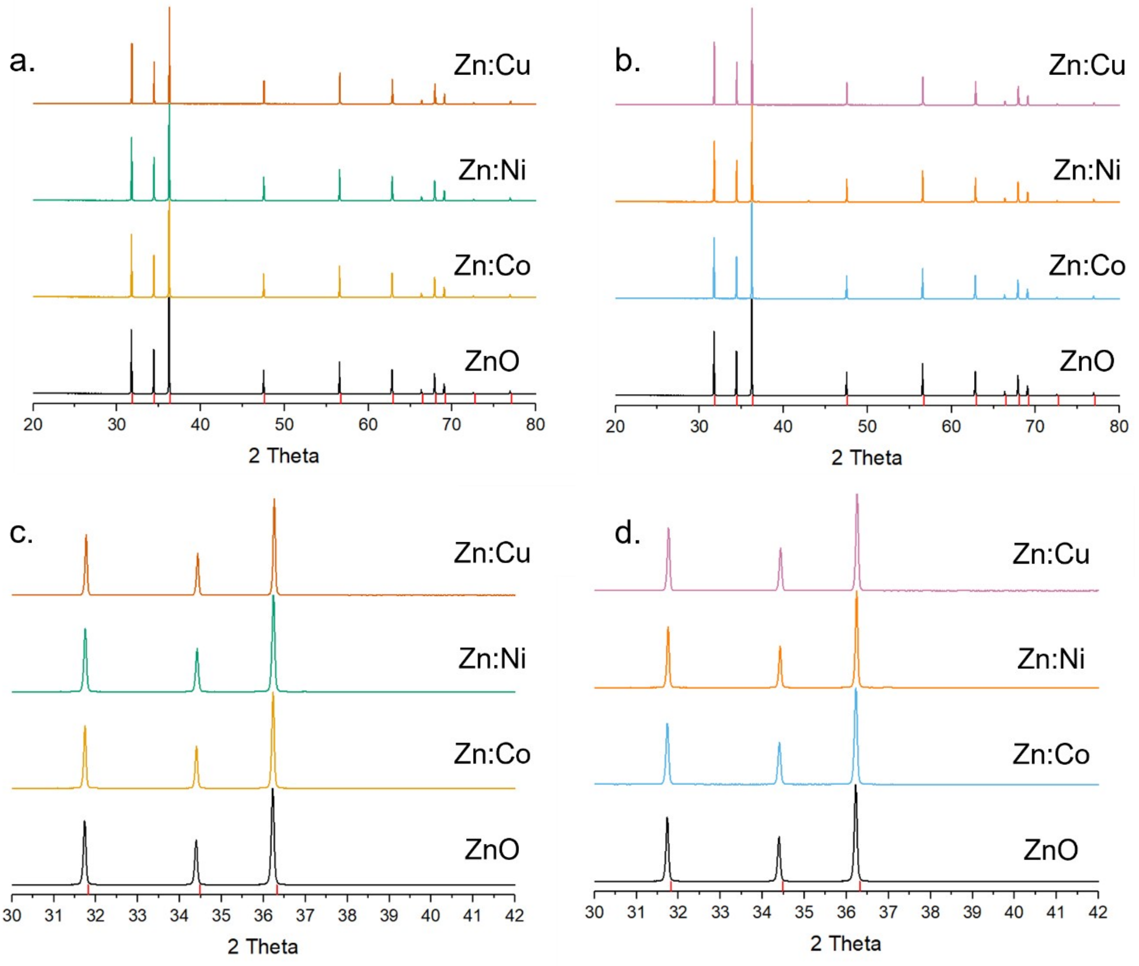

Figure S2: XRD spectra of a,c) 1% and b,d) 2% transition metal doped ZnO molfoams. Tick marks correspond to peaks reported from JCPDS No. 36-1451 [1]

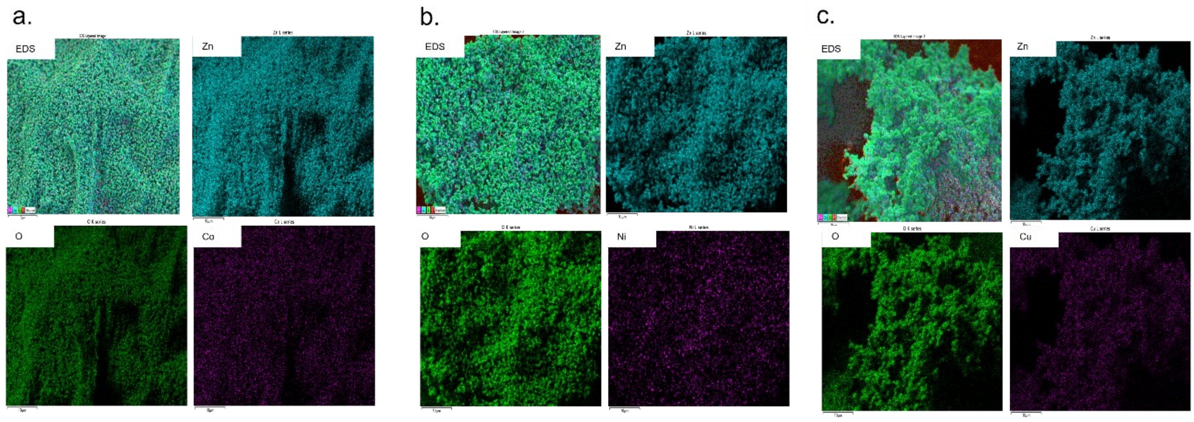

Figure S3: EDX elemental mapping for ZnO doped with a) Co, b) Ni and c) Cu.

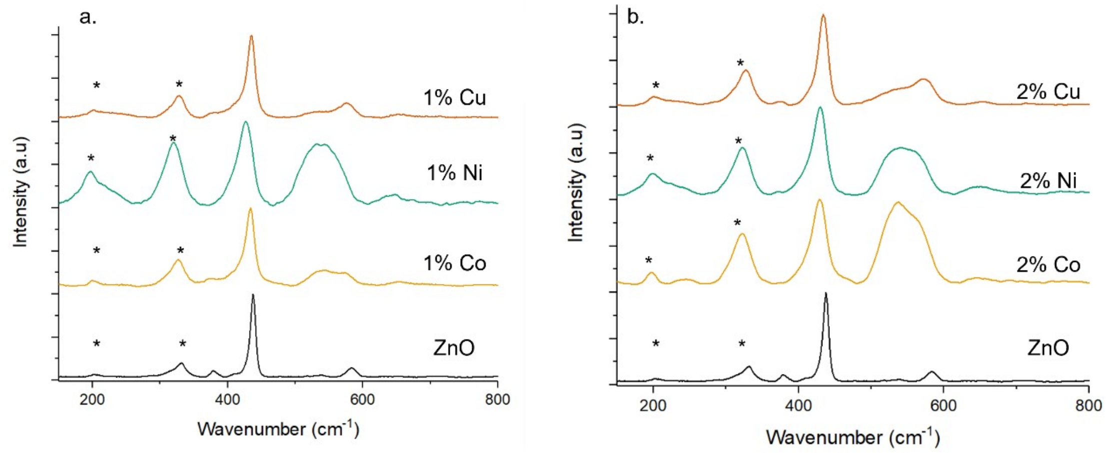

*Figure S4: Raman spectra of pure and doped ZnO molfoams at dopant concentrations of a) 1% and b) 2%. * correspond to multi-phonon features.*

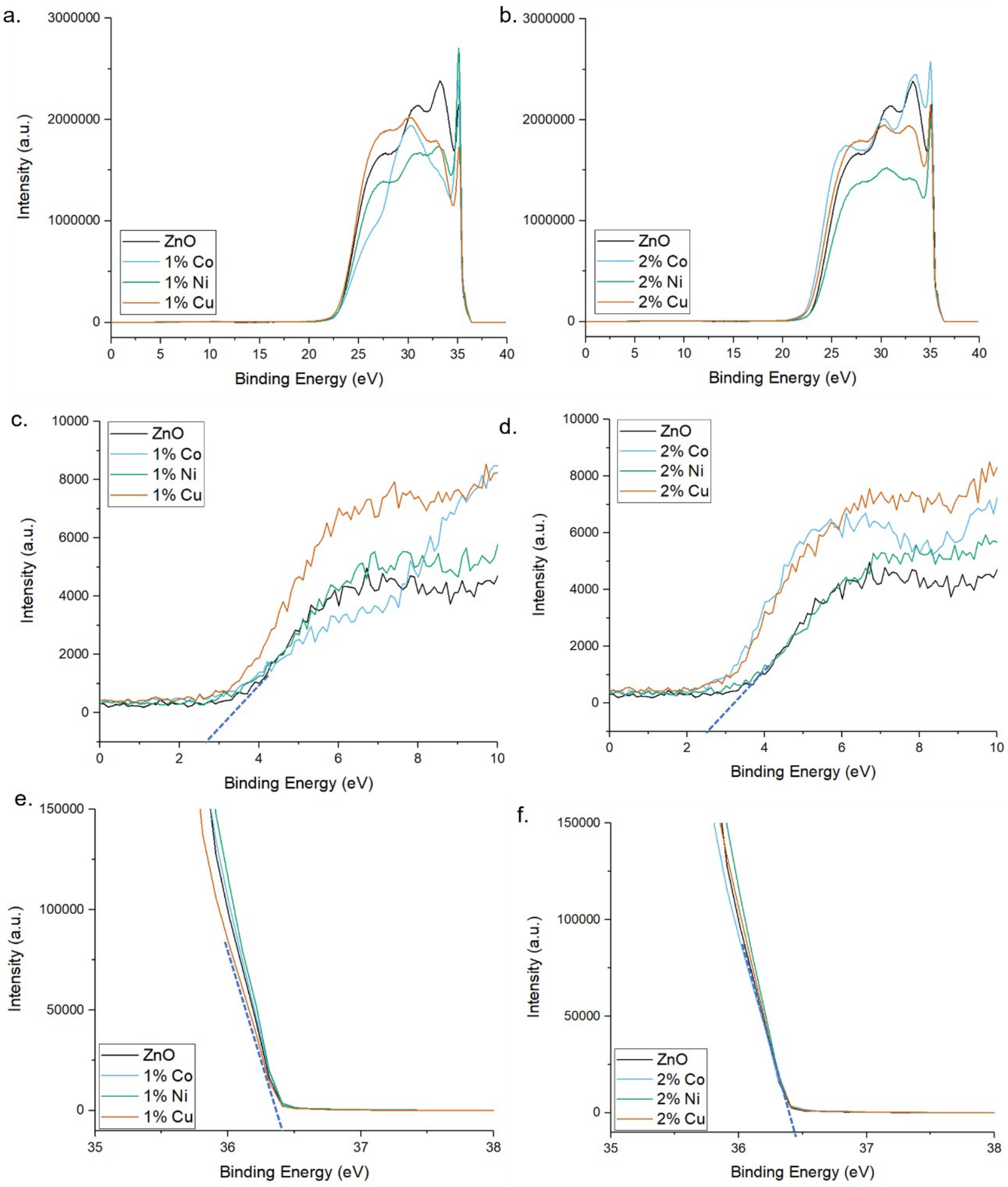

Figure S5: UPS spectra of doped and pure ZnO showing a,b) full spectra, c,d) valance band region and e,f) cut off region.

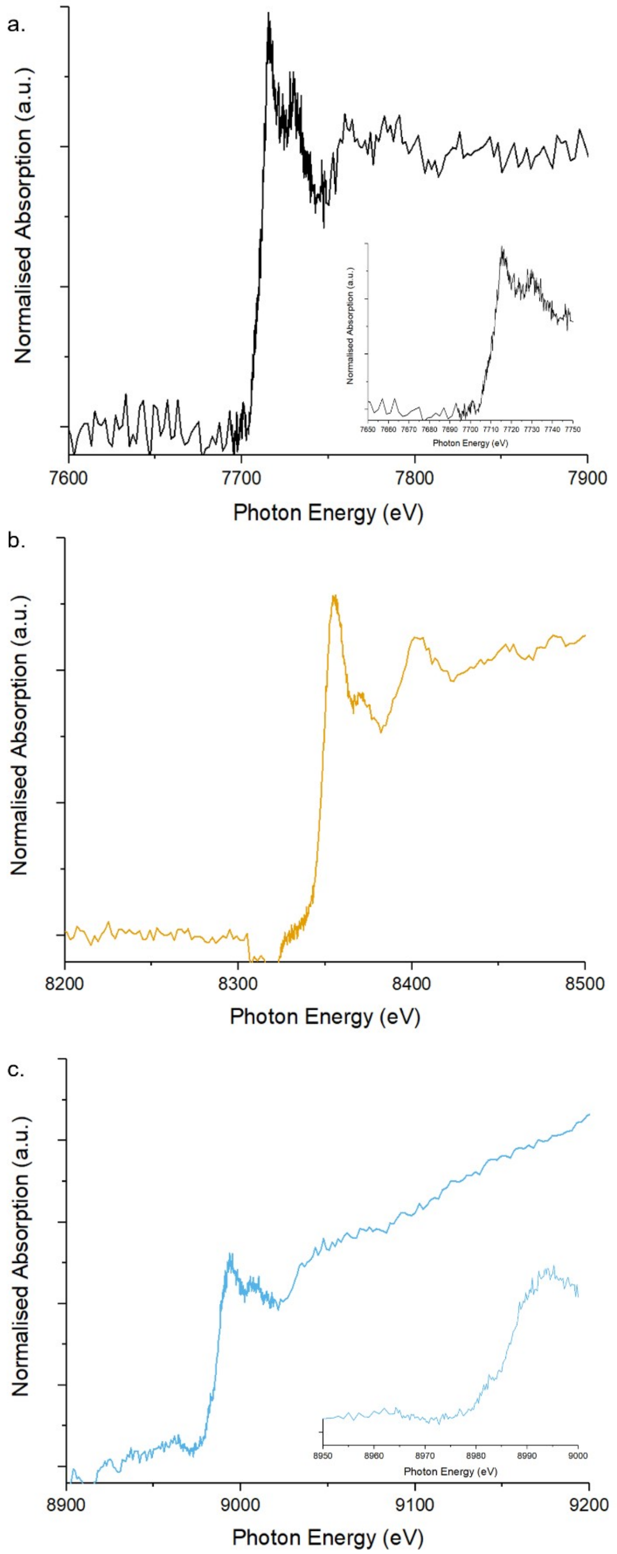

Figure S6: Xanes spectra of a) Co, b) Ni and c) Cu within doped ZnO foams. The insets of a and c show clearer the pre-edge features.

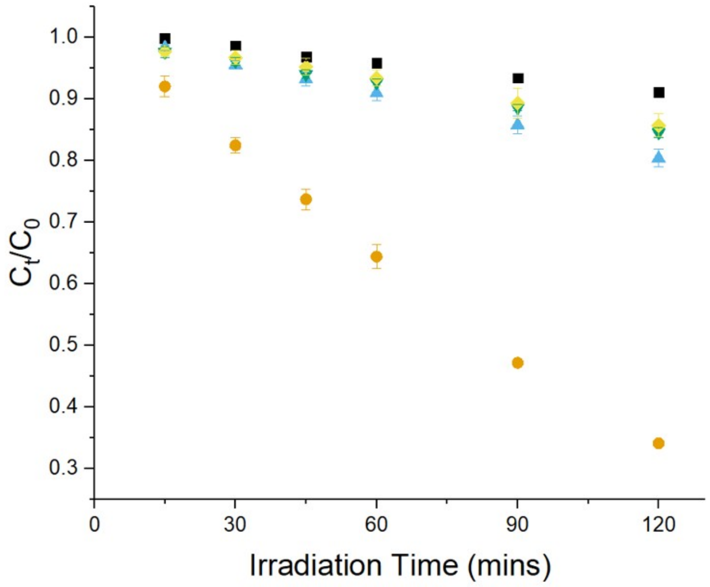

Figure S7: Photocatalytic degradation of CBZ using ZnO doped with various concentrations of Cu:

× *photolysis,* ● *undoped,* ◻ *0.5%,* ❑ *1% and* ◻ *2%.*

Text S1. Quantum yield calculations

The quantum yield allows for an assessment of the photon efficiency, assessing the number of pollutant molecules undergoing degradation relative to the number of photons reaching the catalyst surface [2]. Based on the definitions contained in the IUPAC glossary, the following equations are proposed to calculate the quantum yield of photocatalytic foams:

$$k^{'} = (k)(C_0)(V_{Illuminated})\ \ (mol\ s^{-1}) \qquad (1)$$

$$N_P = \frac{I_{\alpha\lambda} * S * t}{E_P}\ \ (-) \qquad (2)$$

$$q_{n,p} = \left(\frac{N_P}{t}\right)\frac{1}{N_A}\ (mol\ s^{-1}) \qquad (3)$$

$$\phi = \frac{k'}{q_{n,p}}\ (-) \qquad (4)$$

where, k’ is the rate of pollutant degradation (mol s^{-1}), k is the kinetic constant (s^{-1}), C_o is the initial pollutant concentration (mol L^{-1}), $V_{Illuminated}$ is the volume of pollutant irradiated.

The number of photons can be calculated using Equation 1, where $I_{\alpha\lambda}$is the attenuated irradiance of the light source accounting for absorbance of the medium and the pollutant molecule(s) (W m^{-2}) [XX], S is the surface of the sample onto which the light impinges (m^2) and t is the time under irradiation.

$E_p = \frac{h * c}{\lambda}$ (J) is the photon energy at the wavelength emitted by the lamps, where h is Planck's constant, c is the speed of light and λ is the wavelength of light (m) from the lamps. The photon flux is the numbers of photons during irradiation of a mol of photons, where N_A is Avogadro's number (equation 3) . Finally, the quantum yield (φ) is calculated using equation 4.

Text S2. Photocatalytic reactor energy consumption calculations.

To assess the viability of scaling up of the system, the energy consumption of the reactor was accounted for by using the electrical energy per order (E_{EO}), defined as the kilowatt hours of electrical energy needed to decrease the concentration of a pollutant by an order of magnitude (90%) in one cubic metre of solution. [3]

$$E_{EO} = \frac{P * t * I * 1{,}000}{V(log \, {C_0}/{C_t})} \quad (5)$$

Where: P is the total power output of the 3 lamps onto the 12 cm long quartz tube (kW), t is the irradiation time (hrs) V is the volume of reservoir (L) and C_0 and C_t are the initial and final concentrations of pollutants respectively. As the foam occupied only a fraction of the quartz tube, the total power of the lamps, which act on the whole quartz tube, was multiplied by the volumetric fraction occupied by the foam (i.e. foam volume/quartz tube volume), to provide the effective power used for photocatalysis, considering that the contribution of photolysis is negligible. This is rendered necessary by the recirculating nature of the reactor, unlike a simple batch reactor, where the entire reservoir would be irradiated. In the present work, the external diameter of the foam corresponds to the internal diameter of the tube, so that the volumetric fraction is equivalent to the ratio of the foam's length to the total length of the quartz tube: 2 cm/12 cm = 0.17. For the recirculating

MolFoam reactors, three 5 W lamps were used, giving a P value of 15 X 10^{-3} kW, irradiation time was 120 minutes, volume of solution was 0.5 L, and the volumetric fraction 0.17.

Text S3. Calculations used in band edge diagram construction.

In order to construct the band edge diagram, the energies of the valence and conduction bands had to be calculated using a combination of ultraviolet photoelectron spectroscopy (UPS) and UV-Vis spectroscopy.

The UPS measurements determined the work function (W) of the samples (Equation 6)

$$W\ (eV) = 40.8\ eV\ He(II) - cut\ off\ region\ intercept \quad (6)$$

Where W is the work function of the material, 40.8 eV corresponds to the characteristic energy of He (II), and cut off region intercept is the extrapolation of where the higher energy portion of the spectra crosses the x axis as shown in Figure S2 c and f.

From this, the energy level of the valence band maximum can be calculated (Equation 7) using the work function of the sample and the x intercept of the valence band region in the low binding energy region of the spectra as shown in Figure S2 b and e.

$$VB_{max}(eV) = W + x\ intercept\ of\ valence\ band\ region \quad (7)$$

$$CB_{min}(eV) = VB_{max}(eV) - Band\ Gap \quad (8)$$

$$E\ vs\ SHE\ (V) = E\ vs\ vac.\ (eV) - 4.5 \quad (9)$$

In order to compare energy values with the redox potentials of the $^{\bullet}OH/\ H_2O$ and $O_2^{\bullet}/O_2$ couples, energies were converted to the scale of the Standard Hydrogen Electrode (SHE) by subtracting 4.5 V, (i.e. 0 V vs Vac = -4.5 V vs SHE) (Equation 9)

1. R. Boppella, K. Anjaneyulu, P. Basak and S. V. Manorama, *The Journal of Physical Chemistry C*, 2013, **117**, 4597-4605.
2. N. Serpone and A. Salinaro, *Pure and Applied Chemistry*, 1999, **71**, 303-320.

3. J. R. Bolton, K. G. Bircher, W. Tumas and C. A. Tolman, *Pure and Applied Chemistry*, 2001, **73**, 627-637.
